# A COVERED CORONARY STENT FOR ACUTE PERFORATION AFTER A PERCUTANEOUS CORONARY INTERVENTION DUE TO CARDIAC ALLOGRAFT VASCULOPATHY

**DOI:** 10.20471/acc.2024.63.s1.2

**Published:** 2024-03

**Authors:** Mario Udovičić, Hrvoje Falak, Miro Raguž, Ilko Vuksanović, Ante Lisičić, Šime Manola, Irzal Hadžibegović

**Affiliations:** 1 Department of Cardiovascular Diseases, University Hospital Dubrava, Zagreb; 2 School of Medicine of the University of Zagreb, Croatia; 3 Faculty of Dental Medicine and Healthcare, Josip Juraj Strossmayer University, Osijek, Croatia

**Keywords:** *Heart transplantation*, *Coronary artery disease*, *Cardiac allograft vasculopathy*, *Percutaneous coronary intervention*

## Abstract

Cardiac allograft vasculopathy (CAV) is diffuse concentric narrowing caused by intimal fibriproliferation of the coronary arteries in patients after heart transplantation (HTx). It affects almost one third of patients over the period of 5 years, and more than 50% after 10 years following HTx and remains a common cause of late graft failure and mortality. Percutaneous coronary intervention (PCI) can be attempted for focal disease preferably with drug-eluting stents, but the only definite solution is re-transplantation reserved for selected patients with severe CAV. We report a case of a 33- year-old patient with a newly diagnosed CAV, in which a PCI of circumflex coronary artery was attempted, resulting in a coronary perforation treated by the placement of a covered single stent.

## Introduction

Heart transplantation is still the gold standard therapy for the advanced end-stage heart failure refractory to medical treatment. Cardiac allograft vasculopathy (CAV) characterized by intimal proliferation and diffuse concentric narrowing of the coronary arteries is a specific form of coronary artery disease, which develops early after transplantation. It is progressive and accounts for major morbidity and mortality late in the transplant natural history (*1*). No successful treatment for CAV has yet been described and cardiac re-transplantation remains the definitive effective treatment for a selected group of patients with severe form. Percutaneous coronary intervention (PCI) and coronary artery bypass grafting (CABG) are palliative methods reserved for patients with severe proximal discrete coronary lesions of proximal or middle vessels (*2*). However, PCI is associated with high rates of adverse events. Here, we bring a case report of a patient who underwent a PCI for CAV, complicating with a coronary artery perforation, which was successfully treated with a covered stent.

## Case report

A male patient born in 1989 was hospitalised at our Cardiology Department in 2015 due to a terminal stage dilated cardiomyopathy. In 2016, he underwent a successful orthotopic heart transplantation. However, in 2017, the patient was treated for tricuspid valve endocarditis and underwent mechanical tricuspid valve replacement (TVR) requiring a continuous warfarin treatment and a dual-chamber pacemaker shortly afterwards. Continued follow-up was uneventful with the immunosuppressive medication consisting of tacrolimus and mycophenolate mofetil. The patient underwent repeated surveillance coronary angiographies (CAG) with the last one in 2020 before the COVID-19 pandemic, all of which were unremarkable.

**Figure 1:**
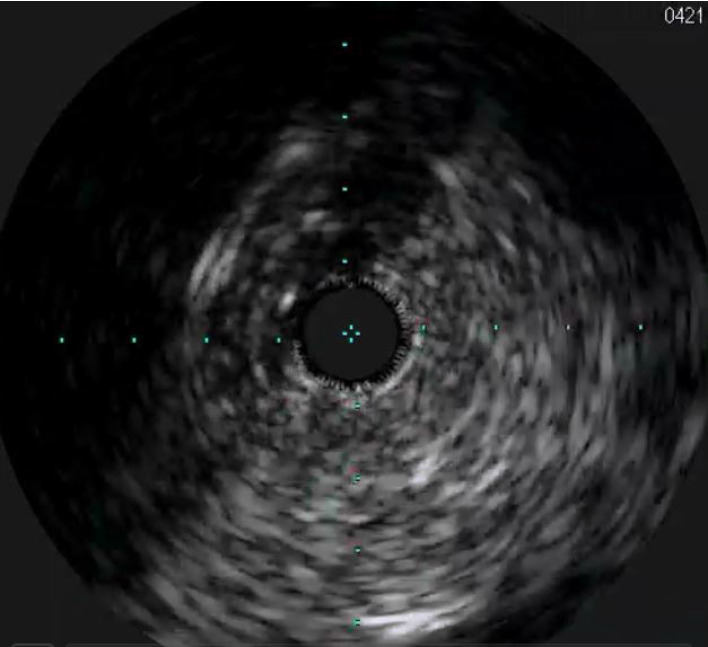
IVUS showing diffuse intimal proliferation and luminal obliteration

In 2022, he was complaining of atypical retrosternal oppressions prompting several visits to the Emergency Department. All findings were within a normal range, and echocardiography also showed no abnormalities with a normal function of mechanical TVR. CAG was repeated using the right femoral approach, as radial approaches could not be established. On the right coronary artery there were insignificant changes in the medial segment and diffuse significant stenoses of the posterolateral branch. On the left coronary artery, diffuse but insignificant changes were noted on the main stem, left anterior descending artery and proximal segment of circumflex artery (CxA). After marginal artery branching, the distal segment of CxA had a subtotal occlusion. Intravascular ultrasound (IVUS) was preformed and visualised the diffuse intimal thickening confirming the diagnosis (Figure 1). IVUS was performed in the distal segment of CxA reporting a diameter of 3 mm. The CAV was graded ISHLT CAV2 pointing to a moderate disease. Due to the significant changes in the area of distal circumflex artery, the heart team decided ad hoc to attempt to re-vascularize the branch.

**Figure 2:**
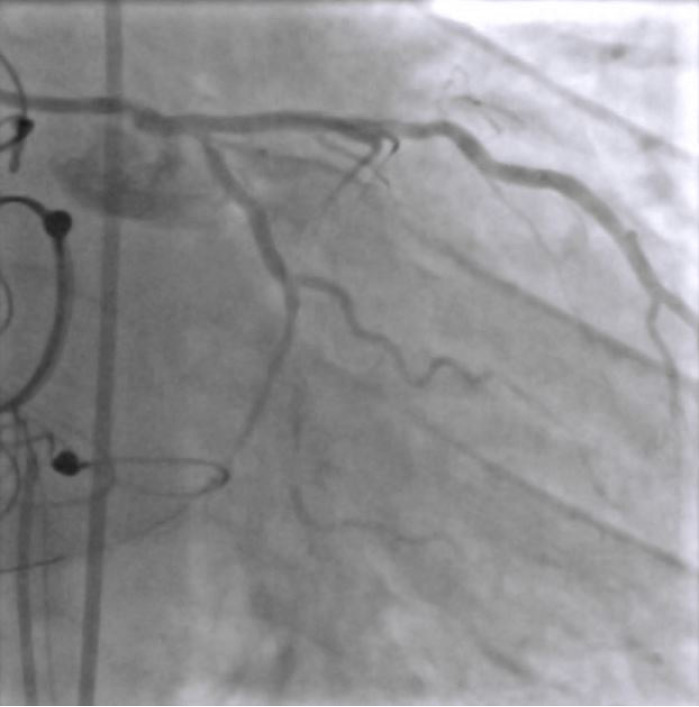
Before PCI - guiding catheter and wire placement in the circumflex artery

**Figure 3:**
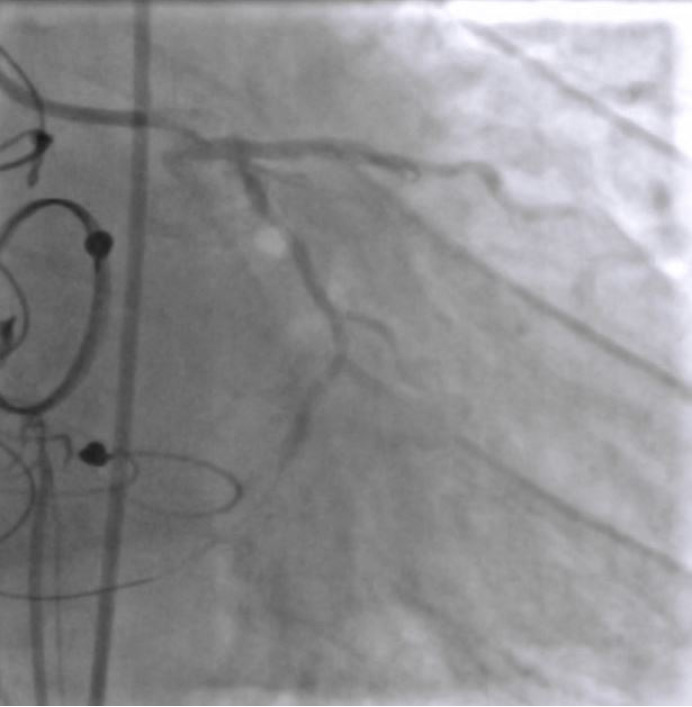
Lesion dilatation with the scoring balloon

**Figure 4:**
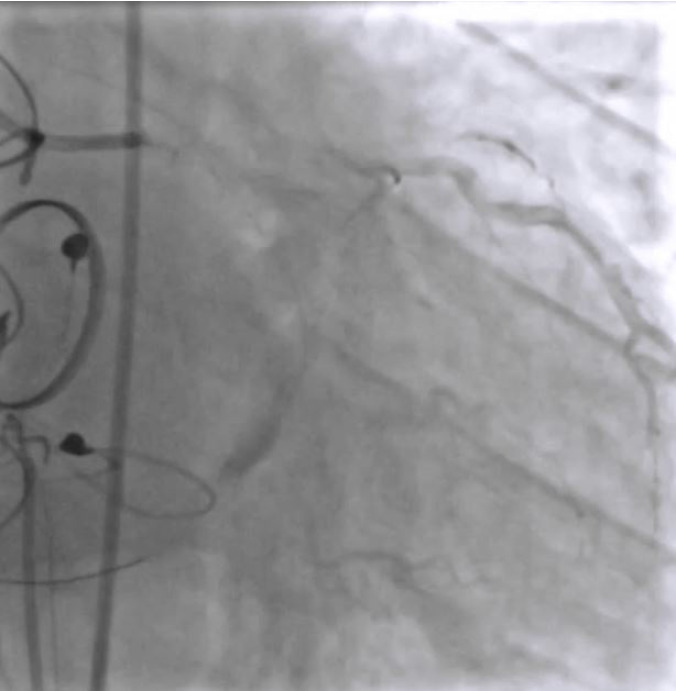
Circumflex artery perforation with extravasation, Ellis type II

**Figure 5:**
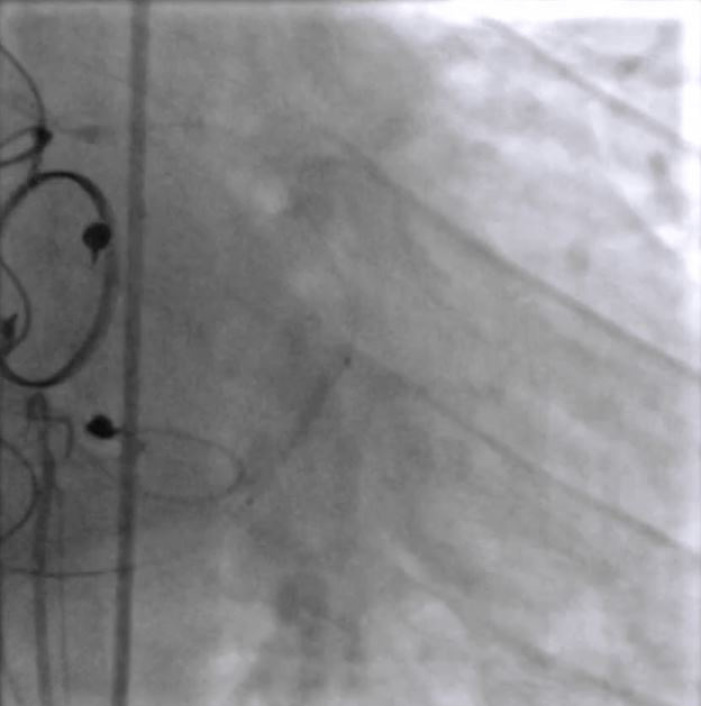
Cover stent placement

**Figure 6:**
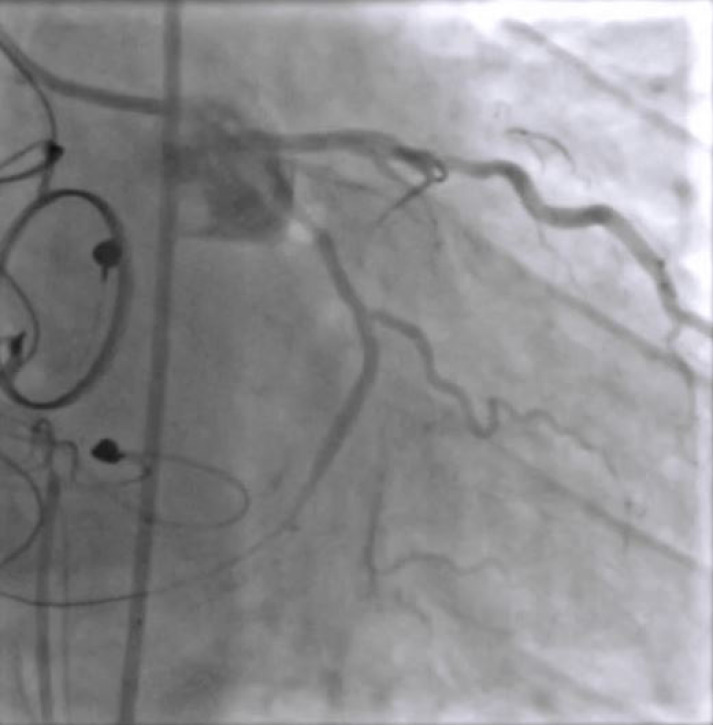
Final result with a good flow through the distal circumflex artery, without extravasation and without residual stenosis

EBU 3.5 6F catheter guiding catheter and workhorse floppy wire were used to negotiate the lesion (Figure 2). Predilatation of the beginning of the distal segment of CxA was attempted with a non-compliant balloon 2.5 x 12, but unsuccessfully due to sliding. Afterwards, the lesion was dilated with a scoring balloon NSE Alpha 2.25 x 13 (Figure 3) but extravasation Ellis type II was shown on the control angiogram (Figure 4). Promptly, the extravasation source was covered by Papyrus stent 2.5 x 20 and the sealing of the origin was then verified (Figure 5). The residual lesion proximally to the cover stent was finally dilated with the drug-coated balloon Sequent Please Neo 2.5 x 15. The final result of the intervention was satisfactory with a flow through the distal CxA, without extravasation and residual stenosis at the place of bifurcation of CxA and the marginal branch (Figure 6).

The patient was in a good condition and discharged five days later. His therapy was modified with statin, calcium channel blocker and acetylsalicilic acid initiated. Two months later, he received a wireless implantable cardioverter-defibrillator for primary prevention, after which mycophenolate mofetil was switched to everolimus. The patient remains well and without any complaints.

## Discussion

CAV is an obliterative and diffuse form of vasculopathy, which is a disease unique to HTx recipients and can be considered a late complication of HTx (*3*). Ischemia is usually clinically silent or subtle due to denervation of the allograft, until the disease is far advanced. The pathological characteristics of CAV differ significantly from those of typical atherosclerotic coronary disease. It consists of concentric and diffuse proliferation of the arterial intima, resulting in thickening and pathological remodelling leading to a progressive narrowing of the lumen, mostly of small and medium-sized arteries (*4*). Invasive coronary angiography still represents the gold standard to routinely screen HTx patients. However, it cannot visualize the arterial wall, which is the reason why IVUS is used to measure accurate luminal diameters and quantify intimal thickening and vessel wall morphology (*1*, *5*).

Treatment options for CAV are not very abundant. PCI and coronary artery bypass grafting are palliative and reserved for patients with a focal stenosis of proximal or middle vessels but the restenosis rates are high. Heart re-transplantation remains the only viable definitive treatment for the end-stage CAV in selected patients.

Our case is the first case report of a coronary artery perforation in a CAV patient treated with a Papyrus cover stent. Unsurprisingly, the PCI in patients with cardiac allograft vasculopathy is associated with higher major adverse cardiac events, including higher rates of in-stent restenosis. There is data indicating that mortality in patients with CAV undergoing PCI is 8 times higher compared to patients managed conservatively, which is associated with an increased length of stay and resource utilizations (*6*) although definitive causality could not be established. Therefore, a careful patient selection and case-to-case decision-making is required with a degree of caution and restraint. The 2021 ACC/AHA/SCAI Guidelines for Coronary Artery Revascularization state that in patients with cardiac allograft vasculopathy and severe proximal discrete coronary lesions, revascularization with PCI is reasonable (*2*).
